# Analysis of Transfer of Tiamulin to Animal Tissue after Oral Administration: An Important Factor for Ensuring Food Safety and Environmental Protection

**DOI:** 10.3390/ph16030387

**Published:** 2023-03-02

**Authors:** Viviana Carmen Ciucă, Carmen Otilia Rusănescu, Victor Viorel Safta

**Affiliations:** Faculty of Biotechnical Systems Engineering, Polytechnic University of Bucharest, 313 Spl. Independentei, 060042 Bucharest, Romania

**Keywords:** tiamulin, withdrawal time, LC-MS/MS

## Abstract

The administration of veterinary medicinal products containing tiamulin hydrogen fumarate (THF) leads to the appearance of the following residues in animal tissues: THF and metabolites that can be hydrolyzed to 8-α-hydroxymutilin. The marker residue for tiamulin, according to Regulation EEC 2377/90, is the sum of the metabolites that can be hydrolyzed to 8-α-hydroxymutilin. The main aim of this study was to analyze the depletion of tiamulin residues and metabolites that can be hydrolyzed to 8-α-hydroxymulinin by liquid chromatography with tandem mass spectrometry (LC-MS/MS) in pig, rabbit and bird tissues after tiamulin administration and to determine minimum withdrawal times for products of animal origin intended for human consumption. Tiamulin was administered orally as follows: 12,000 µg/kg body weight/day for 7 days to pigs and rabbits and 20,000 µg tiamulin/kg body weight/day for 7 days to broiler chickens and turkeys. The values found for tiamulin marker residues were 3 times higher in liver than in muscle in pigs, 6 times in rabbits and 8–10 times in birds. The content of tiamulin residues in eggs from laying hens was below 1000 µg/kg at all times of analysis. The minimum withdrawal times for animal products intended for human consumption, resulting from this study, are 5 days for pigs, rabbits and turkeys, 3 days for broiler chickens and 0 days for eggs.

## 1. Introduction

Monitoring the use of antibiotics in animals is essential to ensure food safety and environmental protection. The administration of veterinary medicinal products containing tiamulin hydrogen fumarate (THF) leads to the appearance of the following residues in animal tissues: THF and metabolites that can be hydrolyzed to 8-α-hydroxymutilin. The marker residue for tiamulin, according to Regulation EEC 2377/90, is the sum of the metabolites that can be hydrolyzed to 8-α-hydroxymutilin. Tiamulin residues in animal tissue can lead to many adverse biological effects and allergic reactions in consumers.

**Figure 1:**
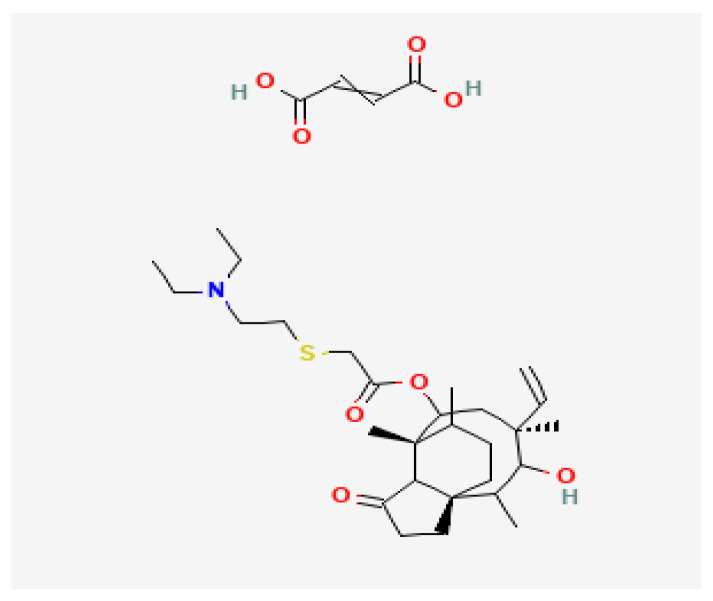
Structure of tiamulin hydrogen fumarate (adapted from [2]).

Tiamulin hydrogen fumarate, discovered in 1950, is a semi-synthetic derivative of the antibiotic pleuromutilin, produced by the basidiomycete *Clitopilus scyphoides* (formerly *Pleurotus mutilis*) and is chemically similar to valnemulin. The mechanism of action of tiamulin consists in inhibiting the synthesis of microbial proteins; this is achieved by binding with rRNA in the peptidyl-transferase slot on the ribosome, in which it prevents the correct positioning of CCA on the ends of tRNA for the transfer of peptides and the subsequent production of specific proteins [1]. Tiamulin hydrogen fumarate has a pleuromutilin chemical structure similar to that of valnemulin HCl. The hydroxyacetyl side chain is replaced by a larger diethylaminoethylthioacetyl moiety. This gives tiamulin hydrogen fumarate better hydrophobicity. The hemi-fumarate moiety provides a stable salt with improved water solubility (Figure 1) [2].

Tiamulin is not used in human medicine. It is used exclusively for animals. Tiamulin has demonstrated in vitro activity against porcine and avian Mycoplasma species, as well as against gram-positive aerobic species (streptococci and staphylococci), gram-positive anaerobes (clostridia), gram-negative anaerobes (*Brachyspira hyodysenteriae*, *Brachyspira pilosicoli*) and gram-negative aerobes (*Actinobacillus pleuropneumoniae* and *Pasteurella multocida*). Tiamulin is effective against mycoplasmas resistant to tylosin and erythromycin.

For veterinary medicinal products, the recommended dose for prevention or metaphylaxis when not associated with treatment is between 2000 and 5000 µg tiamulin as hydrogen fumarate/kg b.w. for 5 days to 6 weeks and for treatment-related metaphylaxis it is between 4000 and 10,000 µg for up to 10 days.

Analysis of the distribution of tiamulin in animal tissue is essential for ensuring food safety as well as preventing the spread of resistant bacteria and the acquisition of bacterial resistance. 

The withdrawal time required for safe consumption of meat products from birds treated with tiamulin at therapeutic levels is 72 h in several states of the European Union. No waiting time is required for the removal of tiamulin residues from the egg in several states of the European Union [1,3]. 

In one study, tiamulin residues in meat were determined by liquid chromatography/mass spectrometry. Extraction of tiamulin was performed with acetone-tetrahydrofuran. The organic layer, separated from water with dichloromethane, was evaporated to dryness and the dry residue was diluted in methanol-1-heptane sulfonic acid. The method is advantageous due to the low quantification limit of 0.002 µg/g [4]. Tiamulin content was also successfully determined in medicated feed using a different gradient of 0.1% formic acid in acetonitrile and 0.1% formic acid in ultrapure water and a biphenyl column [5]. 

Studies on the metabolism of tiamulin have resulted in the following findings. Approximately 85–90% of the drug is absorbed. Maximum blood levels were reached 2–4 h after its oral administration. The target tissue for residues is the liver. The antibiotic is rapidly absorbed and the metabolites are eliminated through bile in feces (2/3) and urine (1/3). Only 0.3 to 0.5% of the parent compound is excreted unchanged in the urine. At least 25 metabolites have been found in urine and bile, of which 16 have been structurally identified. In animals, tiamulin is transformed by N-dealkylation, hydroxylation, oxidation and sulfoxidation. Most metabolites have a substantially lower antibacterial activity and the others have no antibacterial activity. In pigs, tiamulin is readily absorbed and extensively metabolized. None of the 14 metabolites found in pig urine exceeded 6% of the dose and none of the 16 metabolites in pig bile exceeded 7% of the administered drug dose. All individual metabolites found in pig urine, bile or liver (target tissue for residues) represent up to 10% of the administered dose [6]. 

The distribution of tiamulin in animal tissues was studied by gas chromatography with electrochemical detection in pigs and turkeys, by liquid scintillation counting (LSC) in broilers and by gas chromatography (GLC) with an electron capture detector in rabbits. Under the same treatment conditions, similar results were obtained by the LC-MS/MS method described in this study, an equally efficient and accurate method [7,8,9,10,11,12,13,14,15,16,17,18,19].

The main aim of this study was to analyze the depletion of tiamulin residues and metabolites that can be hydrolyzed to 8-α-hydroxymulinin by an effective method, LC-MS/MS, in pig, rabbit and bird tissues after tiamulin administration and to determine minimum withdrawal times for products of animal origin intended for human consumption, according to European Medicines Agency (EMEA) requirements and EEC Regulation 2377/90 [20,21]. Compliance with the withdrawal period for residues to reach concentrations below the limits of safety protects humans from exposure to medicinal substances added to food. It is the responsibility of veterinarians and animal breeders to follow and comply with the withdrawal period in treated animals. 

## 2. Results

### 2.1. Calculation of the Recovery Percentage from Samples

The calibration curve was drawn with the standard calibration solutions (0.05; 0.1; 0.25; 0.5; 1.0 μg/mL) in the control sample extract (matrix), which under working conditions (2 g sample/1 mL final purified extract) corresponds to 25–500 µg/kg; the linearity of the response was checked, then the percent recovery of tiamulin was calculated.

### 2.2. Calculation of Residue Content

The residue concentration was calculated using the calibration curve, taking into account the percentage recovery, or using the following formula:

(1) $$Tiamulin(8\text{-}\alpha \text{-}hydroxymutilin)=c_{pr}\cdot \frac{V_{extract\;final}\;}{m}\cdot \frac{100}{R}\cdot 1000\;\mu g/kg$$

where *c_pr_* is the concentration of tiamulin (8-α-hydroxymutilin) in the final sample extract, read from the calibration curve, in μg/mL; *V_extract final_* is the final sample extract volume, in mL; *m* is the mass of sample taken in work, in g; and *R* is the recovery percentage.

### 2.3. Quality Control

A negative control without traces of tiamulin and a positive control obtained by fortifying the control sample to maximum residue limits (MRLs) were added to each series of samples analyzed.

### 2.4. Identity Confirmation

The ESI-MS/MS technique performed with the acquisition of the spectrum is good evidence of the identity and quantification. The spectrum obtained from the extract must match the spectrum of the standard solution analyzed in the same series of samples. The base ion must be the same. Qualifier ions must be present and have a relative abundance comparable to that of the standard.

The precursor ion and two MRM transitions corresponding to two fragmentary ions were selected, which means 4 identification points (Table 1 of Directive 2002/657/EC) [20,22]. The identity confirmation is presented in Table 1.

The results obtained for tiamulin hydrogen fumarate (THF) marker residues are shown in Table 2, Table 3, Table 4, Table 5 and Table 6. The values shown are corrected with recovery coefficients. The content of tiamulin and 8-α-hydroxymutilin for the samples taken from untreated control animals was below the detection limit of the analysis method. 

The highest concentrations of tiamulin (and 8-alphahydroxymutilin) residues were found in the liver in all species tested. In turkeys, at 7 days after treatment (20,000 µg tiamulin/kg body weight/day), the residues in the liver samples were below 100 µg/kg. 

The residues from pig liver (608 µg/kg) were about three times higher than those from muscle. In rabbits, the residues in the liver (425 µg/kg) were about six times higher than those in the muscles and in birds (chicken and turkey broilers) about four times larger in liver than in muscle and skin/fat. Tiamulin residues in the analyzed eggs were below the MRL (1000 μg/kg) at all times of analysis.

**Figure A1:**
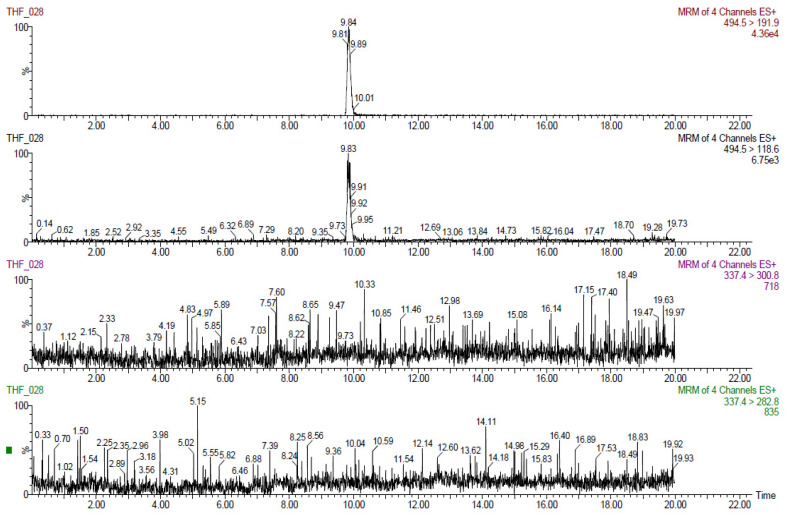
Chromatogram of a muscle sample fortified with tiamulin at 25 µg/kg.

**Figure A2:**
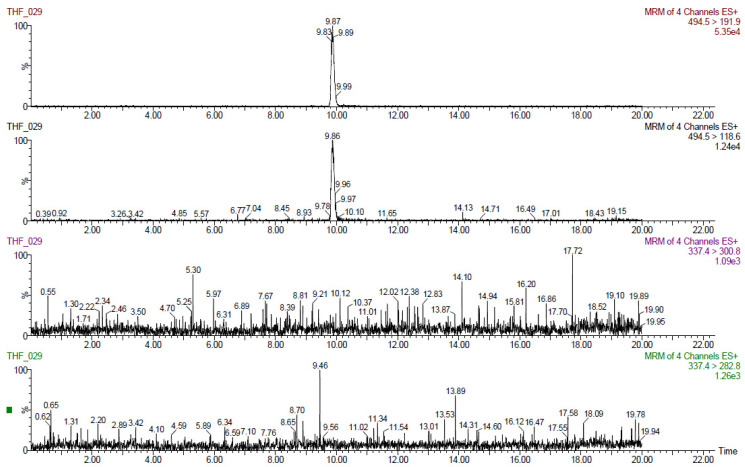
Chromatogram of a liver sample fortified with tiamulin at a level of 25 µg/kg.

**Figure A3:**
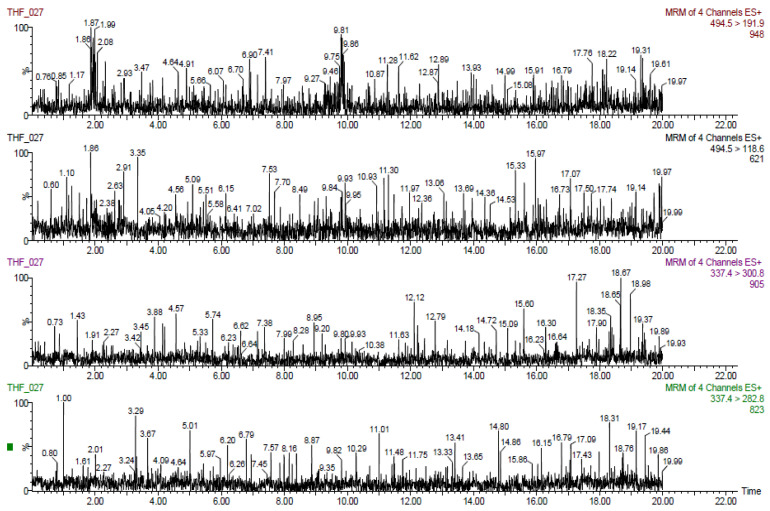
Chromatogram of a blank liver sample.

**Figure A4:**
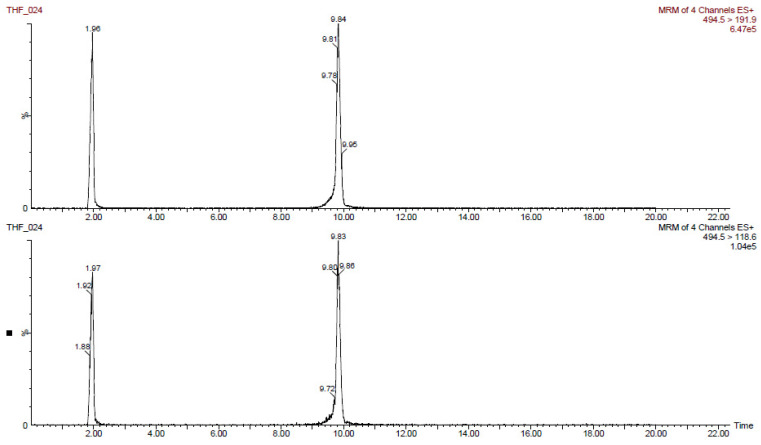
Chromatogram of the reference solution with a concentration of 0.5 µg tiamulin/mL in the matrix.

The concentration of tiamulin (and 8-alphahydroxymutilin) in samples from untreated control animals was below the detection limit of the analytical method (Appendix A, Figure A1, Figure A2, Figure A3 and Figure A4) [23,24,25].

## 3. Discussion

Tiamulin use in intensively raised animals can lead to the appearance of residues in tissues and other products of animal origin intended for human consumption, in higher concentrations than those allowed. The presence of tiamulin in animal products intended for human consumption can often lead to the removal and destruction of a significant amount of meat, causing a serious economic consequences. For this reason, monitoring tiamulin residues is necessary.

For the analysis of tiamulin residues in animal tissues, an analytical HPLC-MS/MS method was developed in this paper. The presented method ensures specificity and selectivity for the determined compounds and efficient recovery and involves simple and fast sample preparation. The precision and accuracy of the described method are validation parameters that meet all the requirements for residue analysis. The results obtained for recoveries on samples fortified at the 0.5, 1 and 1.5× MRL levels exceeded the value of 75.0%, and coefficients of variability were lower than 15%. So, for tiamulin, the mean recovery for muscle was 84.2 ± 5.6% with a coefficient of average CV variability of 6.65%; for the liver, the average recovery was 80.5 ± 7.34% with an average CV coefficient of variability of 9.12%; and for the egg, the average recovery was 79.6 ± 8.80% with an average CV coefficient of variation of 11.06%.

In addition to the fragmentations (m/z) specific for tiamulin, the specific fragmentations were also monitored for 8-α-hydroxymutilin. The limit of quantification based on acceptable precision and accuracy (25 µg/kg) is below the MRL for different tissues, which is a necessary condition for the residue analysis methods. Values for MRL according to the EMA documents for the amount of metabolites that can be hydrolyzed to 8-α-hydroxymutilin are the following: in pigs and rabbits, 100 μg/kg in muscle and 500 μg/kg in liver, in broilers, 100 μg/kg in muscle and skin and 1000 μg/kg in liver, in turkeys, 100 μg/kg in muscle and skin and 300 μg /kg in liver and in eggs from laying hens, 1000 μg/kg.

According to a study in pigs, concentrations of liver metabolites that could be hydrolyzed to form 8-α-hydroxymutilin were determined by gas chromatography with electrochemical detection. In this paper, the LC-MS/MS method was used to determine the concentrations of liver metabolites that could be hydrolyzed to form 8-α-hydroxymutilin under the same conditions as in that study. The concentrations detected by gas chromatography with electrochemical detection in pigs with access to feed containing tiamulin at a concentration of 39,000 µg/kg for 10 days were 247 μg/kg 12 h after administration, whereas, in animals fed likewise for 18 consecutive days, mean liver concentrations of 8-α-hydroxymutilin were 184 μg/kg 12 h after administration. Using LC-MS, under the same conditions, mean concentrations of 272 μg/kg and 202 μg/kg, respectively, 12 h after administration were detected.

According to another study, in turkeys that had access to drinking water containing 0.025% *w*/*v* for 5 consecutive days, the concentrations of metabolites that could be hydrolyzed to form 8-α-hydroxymutilin, detected by gas chromatography with electrochemical detection, were less than 50 μg/kg in muscle. The mean liver concentrations of 8-α-hydroxymutilin were 905, 518, 527, 253 and 228 μg/kg at 0 h, 8 h, 1 day, 2 days and 3 days after treatment, respectively. Using the LC-MS/MS method described in this work, under the same experimental conditions, the average concentrations in the liver were 883, 489, 398, 203 and 188 μg/kg at 0 h, 8 h, 1 day, 2 days and 3 days, respectively, after treatment. 

In broilers given 50,000 µg 3H-tiamulin/kg body weight/day for 5 consecutive days, average total residue concentrations in liver, muscle, skin and fat, determined by LSC (liquid scintillation counting), were 108,000, 550 and 6500 μg equivalents/kg, respectively, 2 h after dosing and the average tiamulin residues in liver, fat and muscle were 15,500, 1400 and 2200 μg/kg, respectively. 8-α-hydroxymutilin metabolite residues in broiler tissues represented approximately 7%, 3% and 2%, respectively, of the total residue from the liver, muscle and skin and fat [8,9,10,11].

## 4. Materials and Methods

The method went through the following steps: sample extraction (muscle, liver, kidney) with McIlvaine–EDTA buffer, precipitation of proteins with trichloroacetic acid, purification on an Oasis HLB cartridge and elution with methanol, evaporation of the eluate and reconstitution in 1 mL mobile phase (water–acetonitrile 90:10 *v*/*v* with 0.2% formic acid), LC analysis and MS/MS detection by ESI + technique with MRM monitoring of two transitions characteristic for tiamulin (494.5 > 191.9/494.5 > 118.6) and for 8-α-hydroxymutilin (337.4 > 282.8/337.4 > 300.8) [7].

### 4.1. Reagents Used

The following reagents were used: tiamulin, standard substance (Dr. Ehrenstorfer GmbH—Augsburg, Germany), disodium salt of ethylenediaminetetraacetic acid (Na_2_EDTA dihydrate; Sigma—Livonia, MI, USA), anhydrous citric acid p.a. (Panreac—Barcelona, Spain), pure trichloroacetic acid, 99%, (Fluka -France), disodium phosphate anhydrous (Na_2_HPO_4_) p.a. (Panreac), acetonitrile, methanol, formic acid, 98–100% (Riedel-de Haen—Nottingham, UK), trichloroacetic acid, 20% (*m*/*v*).

The extraction solution is McIlvaine–EDTA buffer 0.1 M at pH 4, stable for a week. Standard stock solutions (1000 µg/mL) in methanol were prepared, stable for 6 months at −20 °C. On the day of use, 5 standard calibration solutions (0.05; 0.1; 0.25; 0.5; 1.0 μg/mL) were prepared in control sample extract (matrix) corresponding to 25–500 μg/kg.

### 4.2. Animal Experiments and Sample Collection

Studies have been carried out on the depletion of tiamulin residues in pigs, rabbits and birds (broiler chickens and turkeys). 

Depletion studies on tiamulin marker residues were performed on: 12 young pigs, weight 25–35 kg, 11 rabbits, weight 2.5–3 kg, 12 broiler chickens, Cob hybrid, average weight 750 g, and 9 turkeys, average weight 2.3 kg.

To eliminate interference, the animals and birds (broiler chickens and turkeys) used in the study were not treated before the experiment with another antibiotic. Birds and rabbits had not previously been treated with an ionophore coccidiostat (monensin, amproli, narasin or salinomycin).

The animals (pigs, rabbits, broiler chickens and turkeys) in the study were divided into two groups: an untreated control group and an experimental group in which the animals were treated according to the scheme of the experimental protocol with an oral solution containing 100,000 µg tiamulin hydrogen fumarate/mL. The administered doses were as follows: in pigs, 12,000 µg tiamulin/kg body/day, for 7 days, in rabbits, 12,000 µg tiamulin/kg body/day, for 7 days, and in birds (broiler chickens and turkeys), 20,000 µg tiamulin/kg body weight/day for 7 days. At specified time points after the end of treatment, animals were sacrificed for tissue sampling (muscle, liver and kidney).

### 4.3. Equipment and Materials

The chromatographic system used in the study was a Waters 2695 high-performance liquid chromatography (HPLC) system (Waters Corporation, Milford, MA, USA), consisting of a vacuum degasser, a binary pump, and an automatic sampler. An analytical Xbridge RP 18 column (2.1 × 150 mm, 3.5 μm) was used for the elution of the analyte. Using a triple-quadrupole mass spectrometer (Quatro micro MS-MS detector Micromass Waters Corporation, Milford, MA, USA) connected to an electrospray ion source; positive MRM was carried out with this mass spectrometer equipped with an ESI interface, MassLynx software.

The following equipment were also used in this study: KERN Abj analytical balance, REAX control vortex stirrer (Heidolph, Germany), Ultrathurax IKA T25, refrigerated centrifuge Centra MP 4R, ultrapure water SG GmbH production system, SPE purification station with pump vacuum (Supelco, Germany), Oasis HLB cartridge (60,000 µg/3 cc), Turbo-Vap evaporator (Zymark, Germany), Moulinex “Moulinette” type mixer, DD55210, 600 W, and Sonorex RK 100 H ultrasonic bath.

### 4.4. Sample Preparation

The collected samples were placed in a knife mixer for fine grinding and stored in a freezer (−20 °C) before analysis. Fresh and frozen samples were analyzed. If not analyzed immediately, the samples can be kept for up to 1 month at −20 °C.

#### 4.4.1. Sample Extraction 

For extraction, approximately 2 g of minced and homogenized tissue was transferred to a 50 mL centrifuge tube with a cap. Then, 10 mL of McIlvaine–EDTA buffer was added and vortexed for 1 min at high speed followed by 10 min on a platform shaker at medium speed, then sonicated for 5 min. After centrifugation at 4000 rpm for 10 min, the supernatant was transferred to a test tube. The extraction was repeated with 5 mL buffer solution. For protein precipitation, 1 mL of 20% trichloroacetic acid solution was added to the combined supernatants, then homogenized for 1 min with a vortex mixer and centrifuged for 10 min at 4000 rpm (1685× *g*) at room temperature.

#### 4.4.2. Purification

After preconditioning the Oasis HLB cartridge with 6 mL methanol and 6 mL water, the sample supernatant was transferred to the cartridge. The cartridge was washed with 6 mL water methanol (5 + 95, *v*/*v*) and dried under air current for 10 min. Elution was done with 6 mL of methanol. After evaporating the eluate to dryness under a stream of nitrogen, at 40 °C, the residue, taken up in 1 mL mobile phase water–acetonitrile (90 + 10, *v*/*v*) with 0.2% formic acid, was analyzed by LC-MS/MS.

### 4.5. LC-MS—MS Analysis

#### 4.5.1. LC Working Parameters

The mobile phase consisted of formic acid 0.2% (A) and acetonitrile with 0.2% formic acid (B). Gradient elution was conducted as indicated in Table 7.

The flow rate was 0.2 mL/min and the injection volume was 20 µL. The temperature of the column thermostat was 30 °C. 

Mass spectrometry detection was operated in positive ESI mode with multiple reaction monitoring (MRM) at two specific transitions: for tiamulin, 494.5 > 191.9/494.5 > 118.6, and for 8-α-hydroxymutilin, 337.4 > 282.8/337.4 > 300.8.

#### 4.5.2. MS Analysis Parameters 

The mass spectrometry detection was operated in the positive ESI mode with multiple reaction monitoring (MRM). The operating parameters were set as follows: desolvation gas flow (nitrogen)—400 L/h, dissolving temperature—400 °C and ionization source temperature—120 °C. Fragmentation of molecular ions was done in the collision cell with argon (3.0 × 10^−3^ mbar). Tuning of the device was performed with standard solutions (concentration 10 µg/mL), by direct infusion (flow rate 10 µL/min). The retention time and optimal parameters for tiamulin hydrogen fumarate and 8-α-hydroxymutilin are given below (Table 8).

Voltage at the extractor was 1 V and capillary voltage was 3.5 kV. The evaluations were done with the QuanLynx V4.1 program.

### 4.6. Method Validation

Method validation and measurement uncertainty calculation for the determination of tiamulin residues in tissues by LC-MS/MS were performed in accordance with Food and Drug Administration (FDA) and European Medicines Agency (EMA) guidance requirements, with Directive 2002/657/EC and according to specific internal procedure PS-IP-CD-16.

**Figure A5:**
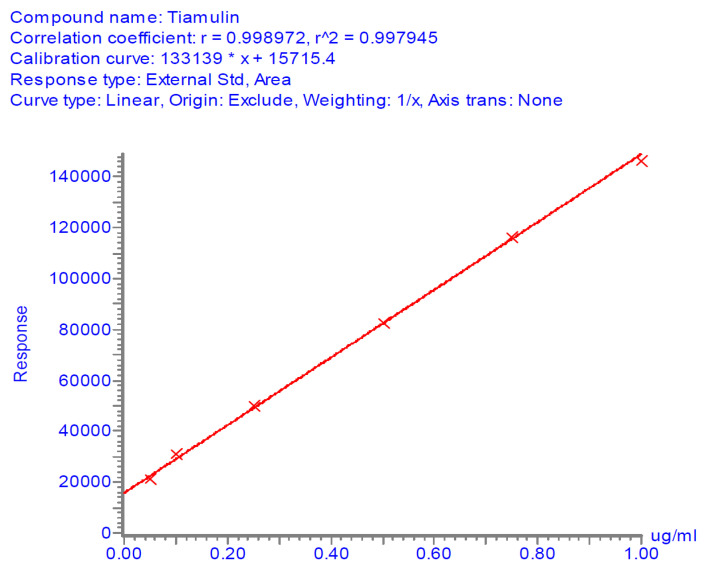
Calibration curve for tiamulin (0.05–1 μg/kg).

All admissibility conditions for all validation parameters (identity, repeatability, reproducibility, specificity, interferences, limit of detection and quantification, linearity, precision and accuracy, decision limit (CCα) and detection capability (CCβ), stability and measurement uncertainty) were met. According to Directives 2002/657/EC [20] and 96/23/EC [22], tiamulin belongs to group B in Annex I, a minimum of 3 identification points being required for the LC-MS/MS technique to ensure the specificity of the method. In our case, the precursor ion and two transitions were selected, corresponding to two fragmented ions, which means 4 identification points [13]. The specific ions chosen for tiamulin were the precursor ion 494.5 and two fragment ions 191.9 and 118.6 and, for 8-α-hydroxymutilin, the precursor ion 337.4 and fragment ions 282.8 and 300.8. The results obtained by comparing the relative intensities of the fragment ions for the samples with those obtained for the standard solution at comparable concentrations, measured under the same conditions, fell within the allowed tolerance limits. In order to determine the linearity of the detector, a series of standard solutions with concentrations between 0.05 and 1.0 µg/mL were analyzed, which corresponds to the conditions described elsewhere in the work (2 g sample/1 mL final purified extract) 25–500 µg/kg (Appendix B, Figure A5). The correlation coefficient (r^2^) was greater than 0.9950. The limits of detection and quantification were calculated by the method based on the calibration curve. The obtained values were LOD = 8.0 μg/kg and LOQ = 25 μg/kg. The limit of quantification was confirmed by fortifying the arrays at the level of quantification obtained and comparing the relative standard deviations with a limit of 20%. No interferences were observed with retention times of tiamulin and 8-α-hydroxymutilin greater than 20% of the response obtained at the limit of quantification of the method. The values found for the relative standard deviations of the repeatability were below the maximum value of 15% established by the legislation. The recovery efficiency and precision of the method were estimated. Control samples treated at the 3 levels, 0.5× maximum recovery limit (MRL), MRL and 1.5× MRL were analyzed in six replicates. The mean recoveries obtained for muscle, liver and egg were 84.2 ± 5.60%, 80.5 ± 7.34% and 79.6 ± 8.8%, respectively, and were within the limits, from 70% to 110%, allowed by legislation [12]. Decision limit (CC_α_) and detection capability (CC_β_) are shown in Table 9.

Quantification of measurement uncertainty was based on precision studies, accuracy studies and the identification and assessment of other contributions to uncertainty.

The calculated values for the expanded uncertainty were between 26 and 36%. The expanded uncertainty of the method was muscle—2 × 0.105 = 0.21 or 21%, liver—2 × 0.132 = 0.264 or 26.4% and egg—2 × 0.145 = 0.29 or 29% [21,22,23,24,25,26].

## 5. Conclusions

The control of antibiotics in the tissues of treated animals is an important factor that ensures the high quality of animal husbandry, consumer protection, as well as environmental protection. A simple and precise method was developed and validated in this study for the determination of tiamulin residues in animal tissue, which is useful for calculating the minimum withdrawal time for animal products intended for human consumption.

Protecting the population from exposure to tiamulin residues in the edible tissues of treated animals involves the determination and observance of withdrawal times. The method for analyzing tiamulin residues by the technique described in this work ensures specificity and selectivity for the determined compounds, fulfills all validation conditions for all analyzed performance parameters and is fast and accurate. By applying the statistical calculation of the European Medicines Agency (EMA) it was found that the minimum withdrawal times for meat and offal from animals treated with an approved oral solution containing 100,000 µg tiamulin hydrogen fumarate/mL are 5 days for pigs, rabbits and turkeys, 3 days for broilers and 0 days for eggs. The withdrawal times obtained as a result of this study fall within the limits stipulated and reconfirmed by Commission Regulation (EU) no. 37/2010. Veterinarians monitor withdrawal times for medicinal products to ensure that illegal levels of residues do not occur in animal products intended for human consumption from treated animals. This study is important because it establishes the withdrawal time and informs the consumer about the risk of consuming meat, organs and eggs that can harm human health.

The withdrawal time calculated and recommended in this study are in accordance with the data published in the specialized literature for products with similar indications of use. The withdrawal times determined in the paper have been confirmed in several European Union states (72 h for birds and zero for eggs) [27,28,29]. 

In the monitoring of tiamulin residues, although the LC-MS/MS method is well designed, demonstrated by its accuracy, sensitivity and precision, it is not without analytical problems. In addition to limitations in selectivity due to the occurrence of “isobaric” interferences, unpredictable attenuations of ion yield, known as the “ion suppression effect”, must be considered. Lack of traceability to reference materials can lead to inaccuracy in testing as well as inaccurate reporting of results if the basic rules of method validation are ignored.

## Figures and Tables

**Table 1:** Confirmation of identity.

Analyte	Precursor Ion (*m*/*z*)	Fragment Ions, (*m*/*z*)	Ion Ratio, (%)	Max. Tolerance Allowed (%)
Tiamulin	494.5	191.9; 118.6	17.6	25
8-alphahydroxymutilin	337.4	282.8; 300.8	3.7	25

**Table 2:** Values found in pigs for tiamulin marker residues (dose 12,000 µg tiamulin/kg body weight/day for 7 days).

Animal Number	Days after Treatment	Muscle (μg/kg)	Liver (μg/kg)
1	1	114	322
2	1	125	488
3	1	95	330
4	1	104	608
5	3	67	112
6	3	41	224
7	3	53	194
8	3	77	334
9	7	38	93
10	7	23	73
11	7	35	51.6
12	7	29	119

**Table 3:** Values found in rabbits for tiamulin marker residues (dose 12,000 µg tiamulin/kg body weight/day for 7 days).

Animal Number	Days after Treatment	Muscle (μg/kg)	Liver (μg/kg)
1	1	54	419
2	1	46.7	425
3	1	72	372
4	1	16.9	120
5	3	12.4	85
6	3	15.5	116
7	3	31.3	141
8	3	10	46.6
9	7	15	92.5
10	7	13.8	65
11	7	11.9	111

**Table 4:** Values found in broiler chickens for tiamulin marker residues (dose 20,000 µg tiamulin/kg body weight/day 7 days).

Animal Number	Days after Treatment	Muscle (μg/kg)	Liver (μg/kg)	Skin/Fat (μg/kg)
1	1	107	622	122
2	1	78	544	118
3	1	88.8	255	127
4	1	91	430	117
5	3	37.5	239	52.8
6	3	44.6	123	41.9
7	3	34.1	119	47
8	3	35	178	55
9	7	25	150	28.8
10	7	18.7	86	21.4
11	7	20	110	18
12	7	16.8	57	16

**Table 5:** Values found in turkeys for tiamulin marker residues (dose 20,000 µg THF/kg body weight/day 7 days).

Animal Number	Days after Treatment	Muscle (μg/kg)	Liver (μg/kg)	Skin/Fat (μg/kg)
1	1	62	400	77.6
2	1	36	356	37.5
3	1	53.5	460	64
4	3	29.5	183	34.2
5	3	17.2	192	31.8
6	3	25.7	226	27.8
7	7	12	88	25.5
8	7	15	80	20.3
9	7	20	110	22

**Table 6:** Content of tiamulin residues in eggs from laying hens (dose 20,000 µg THF/kg body weight /day for 7 days).

Days after Treatment	Residues of Tiamulin in Egg (μg/kg)				
1	412	391	279	366	408
2	552	445	332	519	450
3	469	426	416	387	321
5	269	257	273	166	261
8	129	136	133	185	96
10	100	118	90	72	70
12	90	83	70	50	55

**Table 7:** Mobile phase concentration gradient.

Time (min)	% A	% B
0	90	10
1	90	10
12	66	34
12.5	20	80
15	20	80
15.5	90	10
25	90	10

**Table 8:** Retention time and MS/MS parameters for tiamulin hydrogen fumarate and 8-α-hydroxymutilin.

Component	Retention Time (min)	Precursor Ion (m/z)	Fragmentary Ions (m/z)	Cone Voltage (V)	Collision Energy (eV)
Tiamulin	9.85	494.5	191.9 *; 118.6	26	29
8-α-hydroxymutilin	9.10	337.4	282.8 *; 300.8	24	13

* Quantification ion (most abundant) (dwell time 20 ms).

**Table 9:** Decision limit (CC_α_) and detection capability (CC_β_).

Tissue	Quantity Added (x) (µg/kg)	Quantity Found (y) (µg/kg)	y = ax + b	CC_α_ (µg/kg)	CC_β_ (µg/kg)
Muscle	100	85.2	y = 0.935x − 8.27	93.6	102
Liver	500	388.4	y = 0.668x + 54.4	440.0	491.5
Egg	1000	776	y = 0.696x + 80	929.0	1082

## Data Availability

Not applicable.
